# Supplementary figures and images for: Influence of FGF4 and BMP4 on FGFR2 dynamics during the segregation of epiblast and primitive endoderm cells in the pre-implantation mouse embryo
Source: PLoS One. 2023 Jul 20;18(7):e0279515. doi: 10.1371/journal.pone.0279515 (PMC10358967; doi:10.1371/journal.pone.0279515)

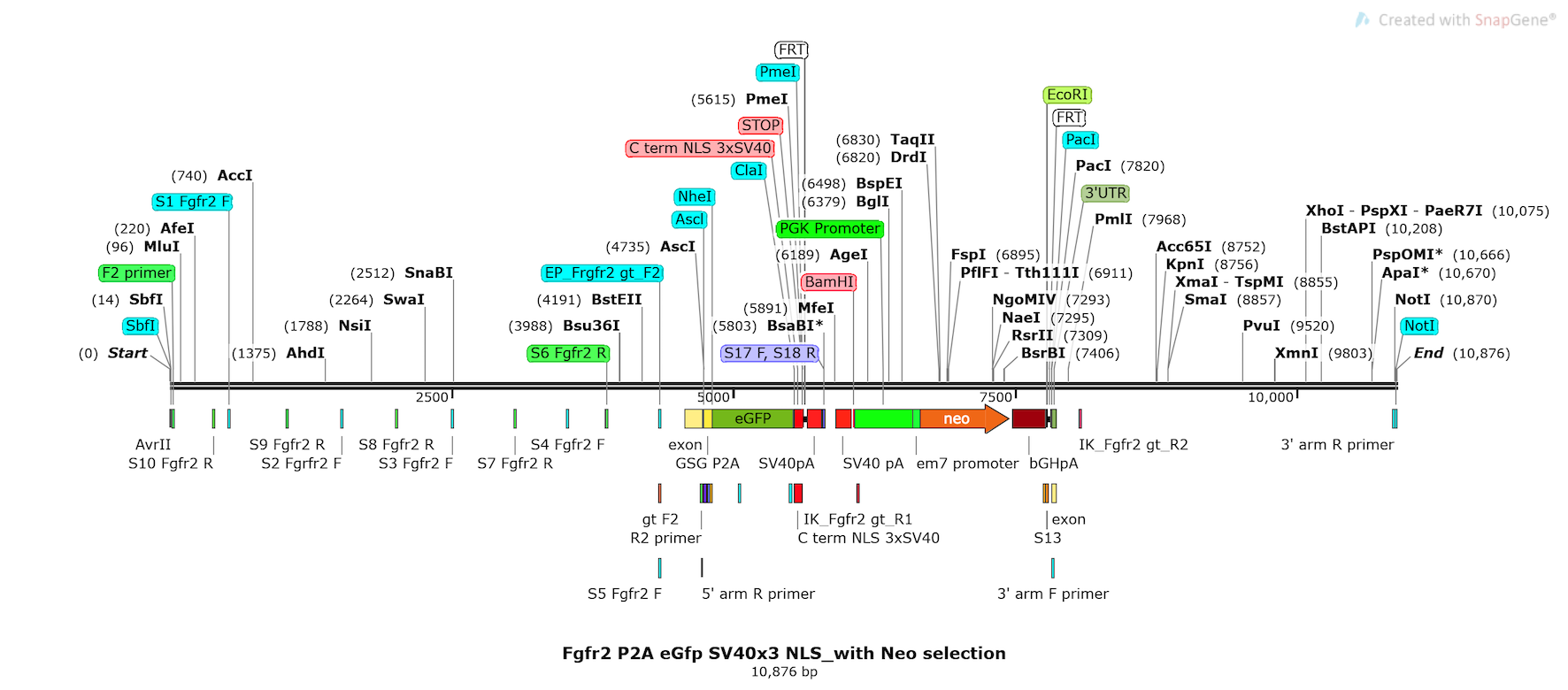

Supplement: S1 Fig — (TIFF) [file pone.0279515.s001.tiff]
